# Inchingorei-san (TJ-117) and Artemisiae Capillaris Herba Induced Prolonged Survival of Fully Mismatched Cardiac Allografts and Generated Regulatory Cells in Mice

**DOI:** 10.1155/2012/689810

**Published:** 2012-07-02

**Authors:** Xiangyuan Jin, Masateru Uchiyama, Qi Zhang, Toshihito Hirai, Masanori Niimi

**Affiliations:** ^1^Department of Surgery, Teikyo University, Tokyo 173-8605, Japan; ^2^Department of Cardiovascular and Thoracic Surgery, The 4th Affiliated Hospital of Harbin Medical University, Harbin 150001, China; ^3^Department of Immunology, Juntendo University Hospital, Tokyo 113-8421, Japan; ^4^Department of Cardiovascular Surgery, Juntendo University Hospital, Tokyo 113-8421, Japan; ^5^Department of Dermatology, Huashan Hospital, Fudan University, Shanghai 200040, China; ^6^Department of Urology, Tokyo Women's Medical University, Tokyo 162-8666, Japan

## Abstract

We investigated Inchingorei-san (TJ-117), a 6-component Japanese herbal medicine, on alloimmune responses in murine cardiac allograft transplantation. CBA mice underwent transplantation of a C57BL/6 (B6) heart and received oral administration of TJ-117 or each component of TJ-117 from the day of transplantation until 7 days afterward. Naive CBA mice rejected B6 cardiac grafts acutely (median survival time (MST), 7 days). CBA recipients given 1 g/kg/day of TJ-117 had prolonged B6 allograft survival (MST, 37 days). Moreover, given 1 g/kg/day of Artemisiae Capillaris Herba (ACH), one component of TJ-117, indefinitely prolonged B6 allograft survival (MST, >100 days). However, other five components of TJ-117 were less effective than TJ-117 and ACH. Secondary CBA recipients given whole splenocytes, CD4^+^, and CD4^+^CD25^+^ cells from primary ACH-treated CBA recipients with B6 cardiac allografts 30 days after grafting had prolonged survival of B6 hearts (MSTs, 57, >100, and >100 days, resp.). Flow cytometry studies showed that the CD4^+^CD25^+^Foxp3^+^ regulatory cell population was increased in transplant recipients given ACH. Cell proliferation, interleukin-2, and interferon-*γ* were suppressed in ACH-treated mice, whereas interleukin-4 and interleukin-10 were upregulated. In conclusion, ACH, one component of TJ-117, as well as TJ-117 induced hyporesponsiveness to fully allogeneic cardiac allografts and may generate CD4^+^CD25^+^Foxp3^+^ regulatory cells.

## 1. Introduction

Transplantation is the ultimate treatment for patients with total loss of function of a life-sustaining organ. New immunosuppressive drugs have improved allograft survival rates; however, all immunosuppressive drugs have specific side effects and additively contributing to an overall state of immunosuppression, which leads to an increased risk of infections and various specific malignant conditions [1, 2]. Although tremendous progress has contributed to the success of this therapy, several challenges remain if transplantation is to be widely available with minimal risks and optimal outcomes. 

Immunologic tolerance is mediated by central and peripheral mechanisms. The mechanisms of peripheral tolerance include anergy, deletion, ignorance and active immune suppression [3]. Among the mechanisms of peripheral tolerance, active suppression by regulatory T cells is likely to have a crucial role in maintaining tolerance to transplants because other mechanisms may not suppress newly generated alloreactive lymphocytes [3]. T regulatory (Treg) cells express transcription factor forkhead box P3 (Foxp3), which is considered the most specific marker to identify the Treg lineage [4–6]. Extensive studies have demonstrated that mutation of Foxp3 or downregulation of Foxp3 expression in Treg cells leads to reduction of Treg cell numbers and loss of Treg suppressive activity and induces immune dysregulation [7–9], strongly suggesting that Foxp3 plays a dominant role in the development and function of Treg cells. Once donor-specific regulatory cells have been induced and survived in the recipient of a graft, it may be possible to modify the life-long use of nonspecific immunosuppressive agents. Therefore, identification of agents that promote induction and maintenance of regulatory cells may have implications for the development of new tolerogenic strategies in transplantation. 

In a murine model, we previously demonstrated the efficacy of the following commonly used agents in inducing donor-specific regulatory cells and prolonging allograft survival: antithrombin III [10], selective cyclooxygenase 2 inhibitor [11], sarpogrelate hydrochloride [12], ranitidine [13], eicosapentaenoic acid [14], ursodeoxycholic acid [15], and danazol [16]. Our recent studies have shown that oral administrations of commonly used Japanese Herbal Medicine, Sairei-to (TJ-114) [17] and Tokishakuyakusan (TJ-23) [18] could significantly prolong survivals of allogeneic cardiac grafts and generate regulatory cells in mice. 

Herbal medicines have been used for over 3,000 years in Asia as alternative therapy for their variety effects and have recently become popular in Europe [19] and the United States [20]. In the last 30 years, Japanese herbal medicines were widely used as alternative therapy for treatment of diseases for their various effects after being recognized officially by Japanese government. For example, Daikenchuto (TJ-100) has been used by gastroenterological surgeons to shorten postoperative ileus after abdominal surgery [21–24], Hochuekkito (TJ-41) has been used to treat atopic dermatitis [25], Hangekobokuto has been used to treat functional dyspepsia [26], and TJ-23 has been used to treat many gynecologic disorders, with few side effects [27]. TJ-23 even could suppress the impairments of lower limbs and exert a favorable effect on cerebral function for poststroke patients [28]. In the current study, we administered other several Japanese herbal medicines individually to mice with fully mismatched cardiac grafts to determine whether any of the agents affected the immune response, and we found that one of these medicines, Inchingorei-san (TJ-117), yielded promising results and was investigated further. 

TJ-117, which is called Yinchen Wuling Powder in China, is composed of 6 herbs: Artemisiae Capillaris Herba (ACH), Polyporus sclerotium, Cinnamomi cortex, Alismatis rhizoma, Atractylodis lanceae rhizoma, and Poria sclerotium. TJ-117 has long been used for the treatment of vomiting, urticaria, liver, and kidney disorder, with few side effects. Recent study showed that TJ-117 had effects in preventing and treating hyperlipoproteinemia [29]. However, there is not still any research of TJ-117 or its 6 components on organ transplantation until now.

 In this study, we investigated the effect of TJ-117 and its 6 components on alloimmune responses in murine cardiac allograft transplantation.

## 2. Materials and Methods

### 2.1. Animals

Male C57BL/6 (B6, H2^b^), CBA (H2^k^), and BALB/c (H2^d^) mice that were 8–12 weeks of age were purchased from Sankyo Ltd. (Tokyo, Japan), housed in conventional facilities at the Biomedical Services Unit of Teikyo University, and used in accordance with the guidelines for animal experimentation approved by the Animal Use and Care Committee of Teikyo University.

### 2.2. Heart Transplantation

All transplant procedures were performed with the mice under general anesthesia. Fully vascularized heterotopic hearts from B6 or BALB/c donors were transplanted into CBA mice by using microsurgical techniques [30]. Postoperatively, graft function was assessed daily by palpation for evidence of contraction. Rejection was defined as complete cessation of the heartbeat and confirmed by direct visualization and histologic examination of the graft.

### 2.3. Administration of TJ-117 and Its 6 Components

Naive CBA recipients of a B6 heart were given no treatment, distilled water (control group), or oral administration of 1 and 0.5 g/kg/day of TJ-117 from the day of transplantation to 7 days afterward. To identify a specific component of TJ-117 responsible for the immunomodulatory effect, other recipients were given 1 g/kg/day of only 1 of the 6 herbal components of TJ-117. All the herbal medicines were dissolved in distilled water and given orally with use of a metal tube (Thomas Scientific, Swedesboro, NJ). The medicines were made as frozen dry powder gifted by Tumura (Tokyo, Japan).

### 2.4. Adoptive Transfer Studies

Adoptive transfer studies were conducted to determine whether regulatory cells were generated after treatment with ACH. Thus, 30 days after CBA recipients (primary recipients) underwent transplantation of a B6 cardiac allograft and were given ACH (1 g/kg/day), splenocytes (5.0 × 10^7^) from primary recipients with functioning allografts were adoptively transferred into naive CBA mice (secondary recipients). After the adoptive transfer, the secondary recipients underwent transplantation of a B6 or BALB/c heart, immediately. In some experiments, CD4^+^ cells were purified from the spleens of primary transplant recipients given ACH by positive selection using a magnetically activated cell sorter (MACS) and CD4 microbeads (Miltenyi Biotec, Auburn, CA; purity >98%), and 2.0 × 10^7^ of the CD4^+^ cells were adoptively transferred into naïve secondary recipients, which then immediately underwent transplantation of a B6 heart. In other experiments, CD4^+^CD25^+^ cells were purified from the spleens of primary recipients given ACH by using MACS and a mouse CD4^+^CD25^+^ regulatory T-cell isolation kit (Miltenyi Biotec). 1.0 × 10^6^ of the CD4^+^CD25^+^ cells were adoptively transferred into naïve secondary recipients, which then immediately underwent transplantation of a B6 heart.

### 2.5. Immunohistochemical and Histologic Studies of Harvested Grafts

Cardiac grafts transplanted into untreated mice and ACH-treated mice were removed 30 days after transplantation and studied immunohistochemically with use of double immunostaining. Fresh 4-*μ*m-thick graft cryosections were fixed in ice-cold acetone and preincubated in Block Ace (Dainippon Pharmaceutical Co., Ltd., Tokyo, Japan). Samples were incubated with anti-Foxp3 (kindly provided by Professor Kenjiro Matsuno [31], Dokkyo Medical University, Tochigi, Japan) polyclonal antibody; incubated with alkaline phosphatase (ALP)-conjugated anti-rabbit Ig (712-055-152; Jackson Immuno Research Laboratories, West Grove, PA, USA) for anti-Foxp3; and developed blue with Vector Blue (Vector Laboratories, Burlingame, CA). Cryosections were then incubated with rabbit anti-mouse type IV collagen polyclonal antibody (LB1403; Cosmo Bio, Tokyo) and peroxidase-conjugated anti-rabbit Ig (55693; Mitsubishi Chemical, Tokyo) and then developed brown with diaminobenzidine (Vector Laboratories).

Cardiac allografts in untreated mice and mice given ACH were removed 30 days after transplantation and studied histologically. Frozen sections (4-*μ*m thick) were cut, mounted on silane-coated slides, and stained with hematoxylin-eosin.

### 2.6. Flow Cytometry Analysis

CD4, CD25, and Foxp3 expression in splenocytes was determined by flow cytometry. Thirty days after cardiac allograft transplantation, splenocytes from recipients treated with ACH and untreated recipients were stained with fluorochrome-conjugated anti-CD4 or anti-CD25 monoclonal antibody (mAb) (RM4-5 and PC61, resp. BD Biosciences, San Jose, CA, USA) and anti-mouse Foxp3 mAb (FJK-16s, eBioscience, San Diego, CA), as well as their isotype controls (eBioscience). The stained cells were analyzed by using a FACS Canto2 system (BD Biosciences). The percentage of CD4^+^CD25^+^Foxp3^+^ in CD4^+^ cells was determined.

### 2.7. Mixed Leukocyte Culture and Cytokine Assays

In mixed leukocyte culture (MLC) studies [32], the responder cells were splenocytes from naïve CBA mice, untreated or ACH-treated CBA mice of a B6 heart 14 days earlier. The stimulator cells were B6 (allogeneic) splenocytes treated with 100 *μ*g/mL mitomycin C (Kyowa Hakko, Osaka, Japan) for 30 minutes at 37°C. The responder cells (2.5 × 10^6^/mL) were cocultured with the stimulator cells (5.0 × 10^6^/mL) in complete medium in a humidified 5% CO_2_ atmosphere (CH-16M, Hitachi, Tokyo, Japan) at 37°C in 96-well, round-bottomed, tissue-culture plates (Iwaki Scitech Division, Tokyo, Japan) for 4 days. Proliferation was assessed by using an enzyme-linked immunosorbent assays (ELISA) for bromodeoxyuridine incorporation (Biotrak, version 2, Amersham, Little Chalfont, UK) according to the manufacturer's instructions [33].

An ELISA was also performed to assess levels of interleukin (IL)-2, IL-4, IL-10, and interferon (IFN)-*γ* in the supernatant of the MLC on day 4. The capture mAb (JES5-2A5), detection mAb (JES5-16E3), and recombinant standard for IL-10 were from BD Biosciences. The capture and detection mAbs for IL-2 (JES6-1A12 and JES6-5H4, resp.), IL-4 (BVD-1D11 and BVD-24G2, resp.), and IFN-*γ* (R4-6A2 and XMG1.2, resp.) were from Caltag Laboratories (Burlingame, CA). Recombinant standards for IL-2, IL-4, and IFN-*γ* were from PeproTech (London, UK).

### 2.8. Statistical Analysis

Cardiac allograft survival in groups of mice was compared by using Mann–Whitney *U* testing (Graphpad Prism, Graphpad, CA, USA). In the cell-proliferation, cytokine studies, and flow cytometry studies, two groups were compared by using unpaired Students' *t*-tests (Graphpad Prism). A value of *P* less than 0.05 was considered statistically significant.

## 3. Results

### 3.1. Survival of Cardiac Allografts in Mice Treated with TJ-117 and Each Component

**Figure 1:**
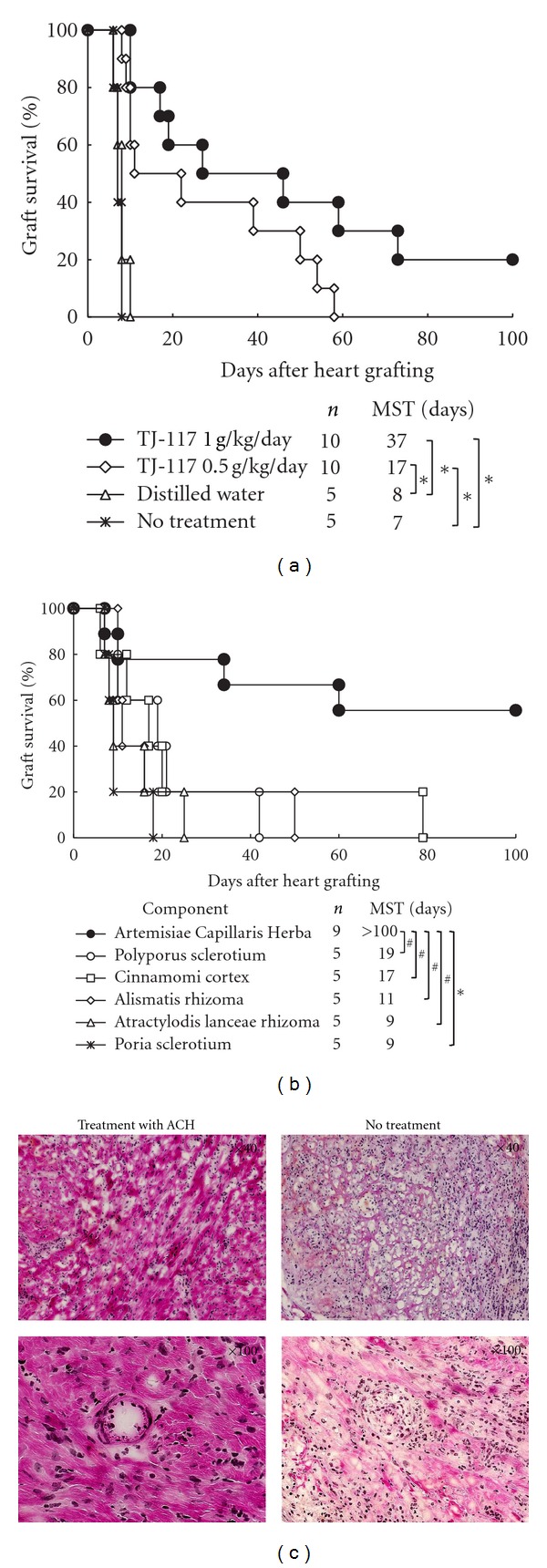
Graft survival of CBA mice given oral administration of Inchingorei-san (TJ-117) and its 6 components and histologic studies. (a) Recipients with C57BL/6 hearts were either untreated, given distilled water, or treated with 1 or 0.5 g/kg/day of TJ-117 from the day of transplantation until 7 days afterward. MST, median survival time; *P < 0.01 for difference between 2 groups. (b) Recipients with C57BL/6 hearts were given oral administration with each component of TJ-117. MST, median survival time; ^#^ *P *< 0.05 and *P < 0.01 for difference between 2 groups. (c) Histologic studies of harvested cardiac allografts stained with hematoxylin-eosin. The left pictures show samples obtained from mice treated with Artemisiae Capillaris Herba (ACH), and the right pictures show samples from untreated mice (magnification ×40 of upper two pictures and ×100 of lower two pictures).

Untreated and treated with distilled water, CBA mice rejected B6 grafts acutely (median survival times [MSTs], 7 and 8 days, resp., Figure 1(a)). CBA recipients given 1 and 0.5 g/kg/day of TJ-117 had prolonged B6 allograft survival (MSTs, 37 and 17 days, resp., *P* < 0.01 versus distilled water-treated and untreated group, resp.).

When CBA recipients received only one component of TJ-117 in a dose of 1 g/kg/day (Figure 1(b)), we found that only treatment with ACH prolonged B6 allograft survival indefinitely (MST, >100 days), while other components induced only modest prolongation on allograft survival.

### 3.2. Histologic Features of Allografts from Recipients Treated with ACH

Histologic examinations of cardiac allografts obtained 30 days after transplantation showed still cell infiltrated but significantly preserved graft structure with a few myocardial injuries and mild obliterative vasculopathy in transplant recipients given 1 g/kg/day of ACH, whereas allografts from untreated recipients showed severe myocyte damage, edema, and aggressive inflammatory infiltrates characteristic of the acute rejection process (Figure 1(c)).

### 3.3. Generation of Regulatory Cells in Mice Treated with ACH

**Figure 2:**
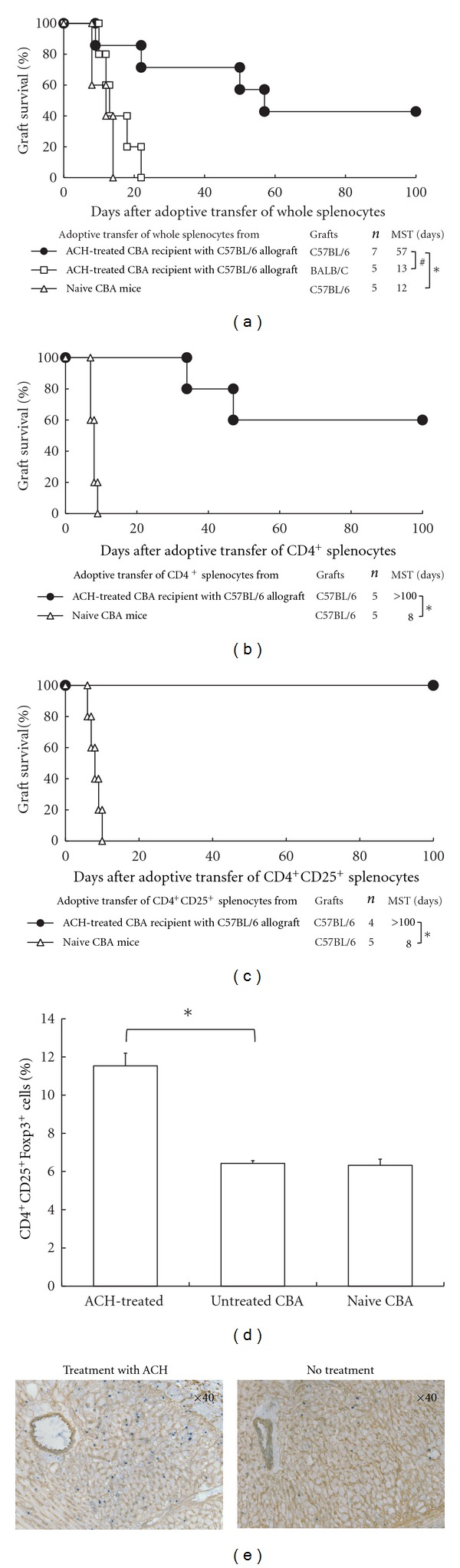
Evidence of generation of regulatory cells in CBA allograft recipients treated with Artemisiae Capillaris Herba (ACH). (a)–(c) Cardiac allograft survival after adoptive transfer of whole splenocytes, CD4^+^, or CD4^+^CD25^+^ cells. MST, median survival time; ^#^ *P *< 0.05 and *P < 0.01 for difference between 2 groups. (d) CD4, CD25, and Foxp3 expression in splenocytes, as determined by flow cytometry. Data are mean ± SD (*n* = 5 mice in each group) for the percentage of CD4^+^CD25^+^Foxp3^+^ in CD4^+^ cells. *P < 0.01 for difference between 2 groups. (e) Foxp3^+^ cells with use of double immunostaining of cardiac allografts obtained 30 days after transplantation from treatment with ACH and untreated recipients (magnification ×40, resp.).

We previously found that some anti-inflammatory or immunomodulatory agents induce hyporesponsiveness to fully allogeneic grafts by means of generation of regulatory cells [11, 12]. To determine whether induction of regulatory cells was involved in the prolongation of allograft survival by ACH, we conducted adoptive transfer studies. Secondary CBA recipients given whole splenocytes from primary ACH-treated CBA recipients with B6 cardiac allografts 30 days after grafting had significantly prolonged survival of B6 hearts compared to secondary recipients, which were adoptively transferred of naïve CBA splenocytes (MSTs, 57 days and 12 days, resp., *P* < 0.01; Figure 2(a)). BALB/c (third party) hearts were eventually rejected in the secondary CBA recipients with the adoptive transfer of whole splenocytes from ACH-treated recipients (MST, 13 days; *P *< 0.05; Figure 2(a)).

When CD4^+^ and CD4^+^CD25^+^ cells were purified from the spleens of primary CBA transplant recipients treated with ACH and adoptively transferred into naïve secondary CBA recipients, the secondary recipients had indefinitely prolonged survival of B6 allografts (MSTs, >100 and >100 days; *P* < 0.01 versus CD4^+^ controls and CD4^+^CD25^+^ controls, resp., Figures 2(b) and 2(c)). In contrast, naïve secondary CBA recipients that underwent adoptive transfer of CD4^+^ (CD4^+^ controls) and CD4^+^CD25^+^ (CD4^+^CD25^+^ controls) cells from the spleens of naïve CBA mice rejected their B6 allografts acutely (MSTs, 8 and 8 days, resp.). These data indicate that treatment with ACH generated regulatory cells which might be donor specific in the primary recipients and that one of the regulatory populations consisted of CD4^+^CD25^+^ cells.

Flow cytometry studies showed that the population of CD4^+^CD25^+^Foxp3^+^ cells in the CD4^+^ cells was increased in the spleens of ACH-treated recipients compared with those of untreated or naïve CBA mice (*P *< 0.01 versus untreated group, Figure 2(d)). The immunohistochemical studies showed that cardiac allografts from ACH-treated recipients had more Foxp3^+^ cells than those from untreated mice (Figure 2(e)). These data suggest that the CD4^+^ regulatory cells contained a population that was CD4^+^CD25^+^Foxp3^+^.

### 3.4. Cell Proliferation and Cytokine Production in Mice Treated with ACH

**Figure 3:**
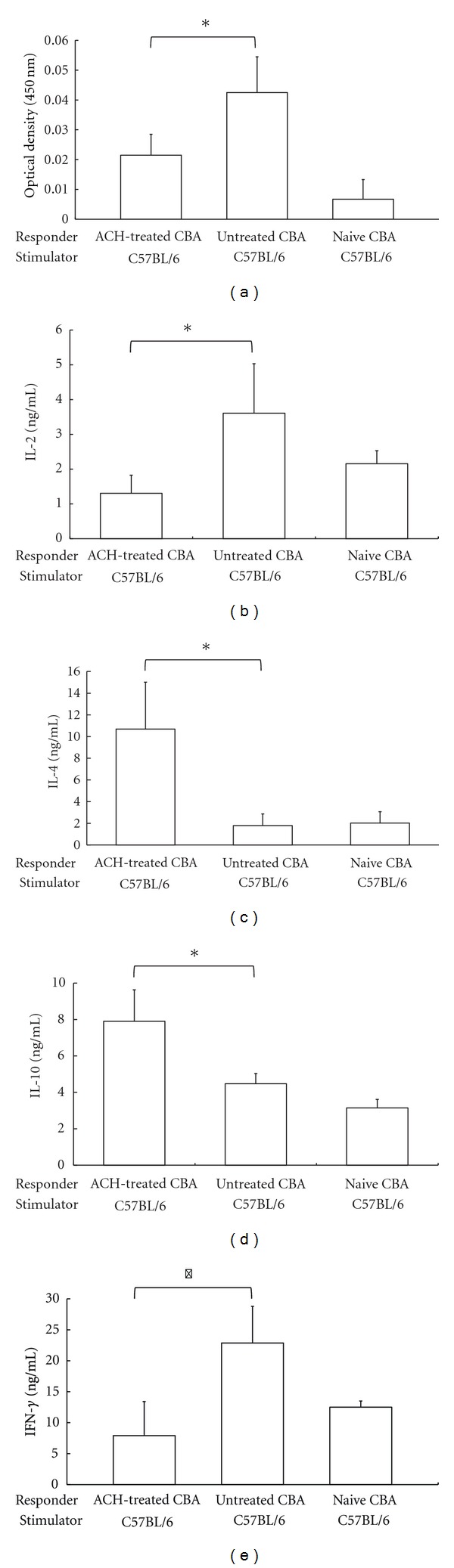
Evidence of induction of alloproliferative hyporesponsiveness by Artemisiae Capillaris Herba (ACH). (a) Results of cell-proliferation assays in mixed leukocyte cultures (MLCs). The data shown are mean ± SD values derived from samples from 5 mice in each group. *P < 0.01 for difference between 2 groups. (b)–(e) Levels of cytokines in MLCs. Levels of interleukin (IL)-2 (b), IL-4 (c), IL-10 (d), and interferon (IFN)-*γ* (e) in the MLCs were assessed by enzyme-linked immunosorbent assays. Data are shown as mean ± SD values derived from samples from 5 mice in each group. *P < 0.01 for difference between 2 groups.

Maximum proliferation of naïve CBA splenocytes (responder cells) against B6 splenocytes (stimulator cells) treated with mitomycin C occurred on day 4 of the MLC. Proliferation of splenocytes from CBA recipients given ACH was significantly suppressed compared with that of splenocytes from untreated mice (*P *< 0.01, Figure 3(a)).

Levels of IL-2 (Figure 3(b)) and IFN-*γ* (Figure 3(e)) in splenocytes from mice treated with ACH were significantly lower than those in splenocytes from untreated CBA mice. In contrast, levels of IL-4 (Figure 3(c)) and IL-10 (Figure 3(d)) were increased in recipients treated with ACH compared with untreated CBA mice.

## 4. Discussion

This study investigated the effect of traditional Japanese herbal medicine TJ-117 on alloimmune responses in a murine model of heart transplantation, and we found that treatment with TJ-117 could induce prolonged survival of fully mismatched cardiac allografts. We also indicated that treatment with ACH, one component of TJ-117, could induce hyporesponsiveness to fully mismatched cardiac allografts in mice. Moreover, treatment with ACH generated regulatory cells that may have been donor specific, and these cells demonstrated suppressive activity in MLC. Furthermore, splenocytes from mice given ACH had downregulated IL-2 and IFN-*γ* and upregulated IL-4 and IL-10 in MLCs.

We considered that there were two possible mechanisms for ACH to contribute to allograft survival. The first one is that treatment with ACH generated regulatory cells. Active suppression by regulatory cells has been found to be one of the important mechanisms of induction and maintenance of self-tolerance [34] and unresponsiveness to allografts [3]. In this study, adoptive transfer of whole splenocytes from primary recipients treated with ACH induced significantly prolonged survival of B6 cardiac allografts in secondary recipients, but secondary recipients rejected the allografts from third-party donors acutely. This result indicates that treatment with ACH generated regulatory cells which might be donor specific, contributing to the prolongation of allograft survival. In addition, adoptive transfer of CD4^+^ or CD4^+^CD25^+^ splenocytes from primary recipients treated with ACH induced indefinitely prolonged survival of allografts in secondary recipients. Moreover, the flow cytometry analysis found that the population of CD4^+^CD25^+^Foxp3^+^cells in the CD4^+^ cells population was increased in transplant recipients given ACH compared with untreated recipients and naïve mice. These results confirmed that the regulatory population generated by ACH contained CD4^+^CD25^+^ cells.

In addition to this possible mechanism for ACH-induced hyporesponsiveness in our model, the balance between Th-1/Th-2 cytokines may have a strong influence on the function of regulatory cells. In our study, expressions of Th-1 cytokines (IL-2 and IFN-*γ*) were decreased and those of Th-2 (IL-4 and IL-10) were increased in ACH-treated mice. In vivo, IL-10 promotes the generation of regulatory T cells [35] and is required for regulatory T cells to mediate tolerance to alloantigens [36]. Moreover, alloantigen-specific regulatory T cells have been shown to prevent rejection initiated by CD4^+^CD25^+^ T cells in organ transplantation [37, 38]. Thus, it is likely that upregulation of IL-10 with ACH resulted in induction of CD4^+^CD25^+^ regulatory cells.

The other one is that ACH had a protective effect on myocardial cells. ACH, one component of TJ-117, has been widely used as a liver protective agent, diuretic, analgesic, lipid digestive agent, antimicrobial agent [39], and a remedy for the treatment of skin inflammatory disorders [40]. The several pharmacological actions of ACH include antiobesity action [41] and liver protective action mediated by antioxidants [42, 43]. In this study, allografts obtained 30 days after transplantation from mice given ACH had only a few myocardial injuries with infiltrating leukocytes and mild obliterative vasculopathy, whereas allografts from untreated group had severe myocardial injuries and obliterative vasculopathy as shown in our histologic examinations (Figure 1(c)). Moreover, because IFN-*γ* could increase expression of class II antigens on endothelial cells [44] and was found to be a key effector in cardiac graft arteriosclerosis [45], we suppose that treatment with ACH seems to have effects of protecting myocardial cells directly, which may include suppressing production of Th1- cytokines (IL-2 and IFN-*γ*).

## 5. Conclusion

Treatment with ACH as well as TJ-117, but not other components of TJ-117, induced hyporesponsiveness to fully allogeneic cardiac allografts and may generate CD4^+^CD25^+^Foxp3^+^ regulatory cells.
